# Supplementary material for: Novel genetic loci affecting facial shape variation in humans
Source: eLife. 2019 Nov 26;8:e49898. doi: 10.7554/eLife.49898 (PMC6905649; doi:10.7554/eLife.49898)
Supplement: Transparent reporting form [file elife-49898-transrepform.pdf]

eLIFE
1st Floor
24 Hills Road
Cambridge CB2 1JP, UK
P 01223 855340
W elifesciences.org
T @elife

eLife's transparent reporting form

We encourage authors to provide detailed information *within their submission* to facilitate the interpretation and replication of experiments. Authors can upload supporting documentation to indicate the use of appropriate reporting guidelines for health-related research (see EQUATOR Network), life science research (see the BioSharing Information Resource), or the ARRIVE guidelines for reporting work involving animal research. Where applicable, authors should refer to any relevant reporting standards documents in this form.

If you have any questions, please consult our Journal Policies and/or contact us: editorial@elifesciences.org.

Sample-size estimation

- You should state whether an appropriate sample size was computed when the study was being designed
- You should state the statistical method of sample size computation and any required assumptions
- If no explicit power analysis was used, you should describe how you decided what sample (replicate) size (number) to use

Please outline where this information can be found within the submission (e.g., sections or figure legends), or explain why this information doesn't apply to your submission:

Sample size of each cohort involved in the study was provided in the Method section. We used fixed samples size as described in the material and Result sections. Power analysis described in Result section showed that under the respective sample size and for study-wide significant threshold, our discovery data had over 90% power to detect an SNP effect explaining 0.54% variance for individual phenotypes, and for the study-wide suggestive significant threshold, the explained variance proportion is 0.43%, as mentioned in the paper.

Replicates

- You should report how often each experiment was performed
- You should include a definition of biological versus technical replication
- The data obtained should be provided and sufficient information should be provided to indicate the number of independent biological and/or technical replicates
- If you encountered any outliers, you should describe how these were handled
- Criteria for exclusion/inclusion of data should be clearly stated
- High-throughput sequence data should be uploaded before submission, with a private link for reviewers provided (these are available from both GEO and ArrayExpress)

Please outline where this information can be found within the submission (e.g., sections or figure legends), or explain why this information doesn't apply to your submission:

We conducted GWAS and meta-analyses for 78 facial traits to test each of 7,029,494 autosomal SNPs and subsequent replication studies. The effective number of independent facial traits was estimated as 43. Based on this, we adjusted the association p-value as 1.2x10-9 as study-wide significant threshold as described in the Methods and results sections. Replication analyses was conducted for 24 independent SNPs and the p-value for replication was adjusted by Bonferoni correction for multiple testing as described in the Method and Result sections. Phenotype outlier exclusion and SNP quality control were described in the Method section. For the in-vitro cell line experiments, we performed three separate transfections at different times (biological replicates); two replicates of each transfection and two replicates of luminscence measurement (technical replicates). Based on the regulations of the cohorts involved, we cannot make individual genotype data publicly available. However, instead we did make all GWAS meta-analysis summary statistic data for all SNPs and all facial phenotypes publicly available via figshare and will additionally make them available via the GWAS Catalogue after the paper is published. This is all described, with necessary details and website links provided, in the Method section.

eLIFE
1st Floor
24 Hills Road
Cambridge CB2 1JP, UK
P 01223 855340
W elifesciences.org
T @elife

Statistical reporting

- Statistical analysis methods should be described and justified
- Raw data should be presented in figures whenever informative to do so (typically when N per group is less than 10)
- For each experiment, you should identify the statistical tests used, exact values of N, definitions of center, methods of multiple test correction, and dispersion and precision measures (e.g., mean, median, SD, SEM, confidence intervals; and, for the major substantive results, a measure of effect size (e.g., Pearson's r, Cohen's d)
- Report exact p-values wherever possible alongside the summary statistics and 95% confidence intervals. These should be reported for all key questions and not only when the p-value is less than 0.05.

Please outline where this information can be found within the submission (e.g., sections or figure legends), or explain why this information doesn't apply to your submission:

We conducted GWAS and meta-analyses for 78 facial traits to test each of 7,029,494 autosomal SNPs and subsequent replication studies. The effective number of independent facial traits was estimated as 43. Based on this, we adjusted the association p-value as 1.2x10-9 as study-wide significant threshold as described in the Methods and results sections. Replication analyses was conducted for 24 independent SNPs and the p-value for replication was adjusted by Bonferoni correction for multiple testing as described in the Method and Result sections. All details of statistical analysis methods were described in the Method section. Raw data were not presented in figures as the size of the data was large. In the GWAS meta analysis we provided exact p value alongside statistical summary and 95% confidence intervals for the 24 significant (lead) SNPs in Supplementary File 1 and Figure 2-figure supplementary 1C-24C. Via Supplementary File 1: Supplementary Table 7 we provided the statistical summary for the 494 study-wide significant SNPs illustrated in Figure 1. Finally, for each SNP used in the GWAS meta-analysis we provide the effect allele, non-effect allele and for each facial phenotype the effect size aligned to the effect allele with standard error and p-value via fighsare and, after paper publication additionally via the GWAS Catalogue, as detailed with website links in the Material and Method section. Regarding the in-vitro experimental work, for each experiment we identified and applied suitable statistical tests, which we fully described in the Method section.

(For large datasets, or papers with a very large number of statistical tests, you may upload a single table file with tests, Ns, etc., with reference to sections in the manuscript.)

Group allocation

- Indicate how samples were allocated into experimental groups (in the case of clinical studies, please specify allocation to treatment method); if randomization was used, please also state if restricted randomization was applied
- Indicate if masking was used during group allocation, data collection and/or data analysis

Please outline where this information can be found within the submission (e.g., sections or figure legends), or explain why this information doesn't apply to your submission:

The discovery GWAS meta-analysis dataset includes 4 cohorts with a total of 10,115 subjects of European descent. The replication dataset included 3 cohorts with 7,917 multi-ethnic subjects. GWASs or replications were independently conducted separately in each cohort and outcomes were combined via meta-analysis. This is all described in the Material and Result sections. Randomization and masking were not applied in this study.

Additional data files ("source data")

- We encourage you to upload relevant additional data files, such as numerical data that are represented as a graph in a figure, or as a summary table
- Where provided, these should be in the most useful format, and they can be uploaded as "Source data" files linked to a main figure or table
- Include model definition files including the full list of parameters used
- Include code used for data analysis (e.g., R, MatLab)
- Avoid stating that data files are "available upon request"

Please indicate the figures or tables for which source data files have been provided:

Via Supplementary File 1: Supplementary Table 7 we provided the statistical summary for the 494 study-wide significant SNPs illustrated in Figure 1. Based on the regulations of the cohorts involved, we cannot make individual genotype data publicly available. However, Moreover, the full GWAS meta-analysis summary statistics of all SNPs analysed and all facial phenotypes studied have been made available via figshare and additionally will be made available via the GWAS Catalogue after the paper is published, as detailed (including doi and website links) in the Material and Method section.
